# Acute and long-term survival in chronically critically ill surgical patients: a retrospective observational study

**DOI:** 10.1186/cc5915

**Published:** 2007-05-15

**Authors:** Wolfgang H Hartl, Hilde Wolf, Christian P Schneider, Helmut Küchenhoff, Karl-Walter Jauch

**Affiliations:** 1 Department of Surgery, Klinikum Grosshadern, Marchioninistr. 15, LMU Munich, D-81377 Munich, Germany; 2 Institute of Statistics, Akademiestr. 1, LMU Munich, D-80799 Munich, Germany

## Abstract

**Introduction:**

Various cohort studies have shown that acute (short-term) mortality rates in unselected critically ill patients may have improved during the past 15 years. Whether these benefits also affect acute and long-term prognosis in chronically critically ill patients is unclear, as are determinants relevant to prognosis.

**Methods:**

We conducted a retrospective analysis of data collected from March 1993 to February 2005. A cohort of 390 consecutive surgical patients requiring intensive care therapy for more than 28 days was analyzed.

**Results:**

The intensive care unit (ICU) survival rate was 53.6%. Survival rates at one, three and five years were 61.8%, 44.7% and 37.0% among ICU survivors. After adjustment for relevant covariates, acute and long-term survival rates did not differ significantly between 1993 to 1999 and 1999 to 2005 intervals. Acute prognosis was determined by disease severity during ICU stay and by primary diagnosis. However, only the latter was independently associated with long-term prognosis. Advanced age was an independent prognostic determinant of poor short-term and long-term survival.

**Conclusion:**

Acute and long-term prognosis in chronically critically ill surgical patients has remained unchanged throughout the past 12 years. After successful surgical intervention and intensive care, long-term outcome is reasonably good and is mainly determined by age and underlying disease.

## Introduction

Several studies have identified significant improvements in acute (short-term) mortality in the general intensive care unit (ICU) population throughout the past decade [1-10]. However, it is unclear whether advances in acute care can be translated into benefits in terms of long-term prognosis, and whether specific subgroups of critically ill patients may profit to a greater or lesser extent [11].

One possible way to define subgroups of critically ill patients is to classify them according to their length of stay in the intensive care unit (ICU). In the past, prolonged intensive care therapy (mostly related to need for mechanical ventilation) has variously been defined as more than 24 hours, more than 2 days, more than 14 days, or more than 28 days [12]. Unfortunately, the findings of studies examining ICU populations with variable length of stay cannot be compared because the degree of critical illness varies directly with length of ICU stay, and because the magnitude of the latter reflects a progressive selection process (survival of the fittest) [13].

Thus far, only five reported studies [14-18] have examined long-term prognosis in critically ill patients with a particularly long length of stay in the ICU (> 28 days). None of these studies examined variables relevant to long-term survival or survival time, although it is likely that, because of progress made in acute care, the number of patients now entering such a chronic state will rise.

Our aim in the present study was to analyze secular changes in acute and long-term mortality in patients who have undergone ICU therapy of duration in excess of 28 days, and to identify prognostic factors that are relevant to acute and long-term prognosis.

## Materials and methods

### Setting and population

The analysis was conducted in the surgical ICU of the Ludwig-Maximilians University Hospital Klinikum Grosshadern in Munich, Germany, which is a 12-bed ICU that mostly receives postoperative patients from the Hospital. Staffing was exclusively surgical and included two senior, board-certified staff intensivists and nine residents (four to five of them were senior residents with at least one year of experience in surgical intensive care). A 12-hour shift system was used throughout the study, with at least one experienced physician in attendance at all times. The nurse/patient ratio varied between 1:2 and 1:3. ICU organization and management were identical during the period of study, meaning that ICU processes and admission, discharge, do-not-resuscitate order and withdrawal of care policies were consistent over time.

The inclusion period extended from 1 March 1993 to 28 February 2005. The observation period started in 1993, when an electronic chart was initiated in our ICU for local benchmarking and in-hospital information transfer. Survival status in all patients was obtained until 28 February 2007. A variety of new therapeutic strategies such as use of low tidal volumes or strict glycaemic control (for review [19]) were applied successively from 1999 onward.

We conducted a retrospective search of all eligible patients, including all consecutive patients admitted immediately or following a delay after a surgical procedure. Because of their small number, all patients who had not undergone surgery during their present hospital stay or who had been admitted only for medical reasons were excluded. Patients who had not consented to undergo prolonged intensive care were excluded from the analysis. Only patients with an ICU stay of longer than 28 days were included. The retrospective data analysis was approved by the local institutional review board. Baseline data and acute outcomes of the entire patient population treated in our institution between 1993 and 2005, and of a specific subpopulation (patients with an ICU length of stay > 4 days) were recently reported [1,20].

### Data collection

We prospectively collected the following information for each patient: age; sex; admission and discharge dates from the ICU; outcome at ICU discharge; cause of death during ICU stay; primary diagnosis (abdominal disease, thoracic disease [mostly pulmonary malignancy], vascular disease, orthopaedic disease, combined diseases, severe sepsis as previously defined [21], pneumonia as previously defined [22], or peritonitis as previously defined [23]); admission state (emergency admission, readmission, immediate postoperative admission, surgery for a benign disease, curative surgery for a malignant disease, palliative surgery for a malignant disease); Acute Physiology and Chronic Health Evaluation (APACHE) II score during the first 24 hours after admission; maximum APACHE II score during ICU stay; maximal number of failing organs during ICU stay (organ failure was defined according to a modified Goris score [24]); and variables related to ICU therapy (duration of invasive mechanical ventilation, duration of catecholamine therapy, need for renal replacement therapy, number of transfused blood units) or to surgical therapy (number of reoperations).

Readmission was defined as an ICU admission after any preceding ICU admission that occurred during the same hospital stay and that lasted less than four weeks. Days or data from the preceding ICU admission were not used in the analysis, except that the patient's admission state was labelled as readmission. If a patient had already stayed on the ICU for more than four weeks, and if they could be discharged later but had to be readmitted a second time, then the patient was included in the study, but the second stay was ignored in the analysis. Sequential organ dysfunction and maximum organ dysfunction were monitored by daily calculation of APACHE II score, because specific methods (Sequential Organ Failure Assessment) were not yet available in 1993 [25].

### Statistical methods

#### Regression modelling of mortality and time to death data

Effects on acute prognosis were either evaluated by analyzing ICU mortality or time to death after inclusion. This duplicate analysis accounted for confounding effects arising from patient transfer to other ICUs or long-term care units. Furthermore, to identify factors that were exclusively relevant to long-term prognosis, we examined two-year mortality in ICU survivors.

Effects of variables were examined by logistic regression analysis and by nonproportional hazard models. Interactions between certain variables (APACHE II score on admission day, maximum APACHE II score during ICU stay, maximum number of failing organs during ICU stay) were also evaluated. The assumption that the effect was linear in the continuous variables was tested using the smoothed scatter plot approach proposed by Kay and Little [26] or by analyzing the effect of estimated coefficients of design variables (quartiles of the covariate distribution) on mortality or cumulative hazard rate [27]. In case of a nonlinear effect, a logarithmic, exponential, power, or quadratic transformation of the variable was tested. If these approaches failed to fit the data, then the covariate was divided into two classes based on median or quartiles [27].

Variables found to be associated with ICU mortality or two-year mortality (ICU survivors) in the univariate analysis (*P *< 0.20, *P *< 0.01 for interactions [28]) were entered into a stepwise multivariable logistic regression model to estimate adjusted odds ratios and 95% confidence intervals. Statistical significance was defined as *P *< 0.05. Goodness of fit was evaluated using Hosmer-Lemeshow statistics.

Effects of variables on survival time during the first two years after inclusion were initially examined using proportional hazard models. The form of relationship between two-year survival and patient variables, and the validity of the assumption of proportional hazards were investigated using plots based on Schoenfeld residuals [29]. These residuals revealed multiple violations of proportional hazards. Because nonproportional effects occurred in all variables before/after days 130 to 150 after inclusion, the time axis was partitioned by censoring all patients either still at risk at 150 days or who had already died before that time point [29]. Thereby, effects on five-month survival and on two-year survival in 150-day survivors could be analyzed separately. Also, within those two separate analyses we generated time-dependent covariates by creating interactions of the predictors and a logarithmic function of survival time, and included both in a combined model. If any of the time-dependent covariates were significant, then those predictors were considered not to be proportional.

Subsequently, a multivariate nonproportional hazard model with backward stepwise elimination of variables was constructed to estimate adjusted hazard ratios and 95% confidence intervals. Variables with a *P *value below 0.20, time-dependent covariates with a P value below 0.10, and interactions with a *P *value below 0.01 by univariate analysis were entered into the model [28]. Statistical significance was defined as *P *< 0.05.

#### Analysis of long term survival beyond the second year after inclusion

Kaplan-Meier survival analysis was used to describe long-term survival after the second year after inclusion (day 28 of intensive care therapy) and to compare survival rates with those of the German average population [30]. For the latter, an ideal reference population was constructed in which the members were all at the same age, matching the mean age of the comparison group.

#### Data presentation and between-group comparisons

Categorical variables were described as percentage and continuous variables as mean ± standard deviation. A *P *value of 0.05 or less in a two-tailed χ^2 ^test was considered statistically significant.

#### Power analysis

One goal of the study was to evaluate differences in long-term survival between two successive six-year periods. A retrospective sample size calculation [29] indicated that 220 events (number of deaths) would allow detection of a 15% absolute increase in five-month survival rate (after inclusion) among critically ill patients with a presumed five-month survival rate of 40%, at a significance level of 5% and a power of 90%.

The statistical analysis was performed using a SAS Package (SAS version 9.1.3, 2002–2003; SAS Institute Inc., Cary, NC, USA) and an R package (version 2.4.1; R foundation, Vienna, Austria).

## Results

### Clinical results

**Figure 1:**
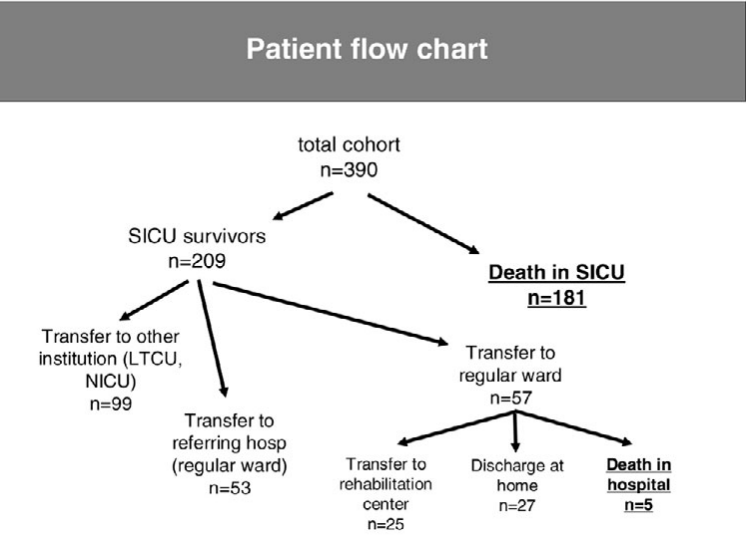
Patient flow after inclusion in the study. LTCU, long-term care unit; NICU, neurological intensive care unit; SICU, surgical intensive care unit.

During the 12-year period of observation, 392 patients had a stay in the ICU stayed of more than 28 days and fulfilled our criteria for inclusion in the cohort. Two patients (0.05%) were lost to follow up and were excluded from the analysis. Clinical data for the whole cohort are presented in Table 1. Surgical ICU length of stay was 62.8 ± 46.4 days, surgical ICU survival rate was 53.6% and 150-day survival rate after inclusion was 42.3%. About half of the surgical ICU patients who had acutely survived their surgical disease were transferred to secondary ICUs in other institutions for weaning after long-term ventilatory support or for neurological/physical rehabilitation (Figure 1). The remaining surgical ICU survivors could be discharged to regular wards and were either directly transferred back to the referring hospital or remained at our institution. Almost half of the latter patients were later transferred to primary/secondary hospitals or rehabilitation centres, whereas most of the remaining patients could be discharged to home.

**Table 1:** Baseline characteristics, clinical variables and variables of intensive care therapy

Variable	Value
Number of patients	390
Age (years)	65.3 ± 13.5 (67.0; 58.0–75.0)
Sex (% male)	71.5
Emergency admission (%)	60.4
Readmission (%)	12.3
Immediate postoperative admission (%)	66.2
Surgical speciality (%)	
Abdominal surgery	48.8
Thoracic surgery	17.7
Vascular surgery	20.8
Orthopaedic surgery	9.8
Combined surgery (%)	1.5
Benign disease (%)	66.3
Curative surgery for malignant disease (%)	21.9
Palliative surgery for malignant disease (%)	11.8
APACHE II score on admission day	18.4 ± 6.9 (18.0; 13.0–23.3)
Pneumonia (%)	68.1
Peritonitis (%)	30.8
Severe sepsis (%)	61.1
Need for mechanical ventilation (%)	99.0
Duration of mechanical ventilation (days)	44.8 ± 44.7 (31.0; 17.0–57.3)
Need for catecholamine therapy (%)	92.3
Duration of catecholamine therapy (days)	28.3 ± 30.4 (18.0; 6.0–32.0)
Need for renal replacement therapy (%)	35.1
Duration of continuous renal replacement therapy (days)	9.8 ± 23.9 (0.0; 0.0–7.8)
Need for red cell transfusion (%)	97.2
Number of transfused red blood cell units	21.8 ± 26.0 (14.0; 6.0–28.0)
Number of surgical revisions	2.1 ± 3.0 (1.0; 0.0–3.0)
Maximum APACHE II score during ICU stay	29.4 ± 6.9 (30; 25.0–34.0)
Maximum number of failing organs	4.4 ± 1.4 (5; 3–6)

Continuous data are presented as mean ± standard deviation (median; 25% to 75% quartile). APACHE, Acute Physiology and Chronic Health Evaluation; ICU, intensive care unit.

### Long-term survival rate

**Figure 2:**
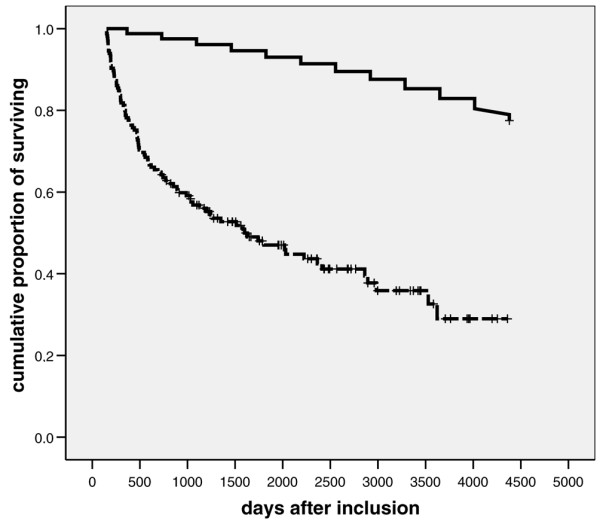
Twelve-year survival: chronically critically ill patients who have already survived 150 days versus general population. Presented are Kaplan-Meier plots showing 12-year survival rates (after inclusion) in patients surviving more than 150 days (dashed line) and in the German general population (continuous line; reference age 61 years; data from Statistisches Bundesamt Wiesbaden, Germany [30]).

**Figure 3:**
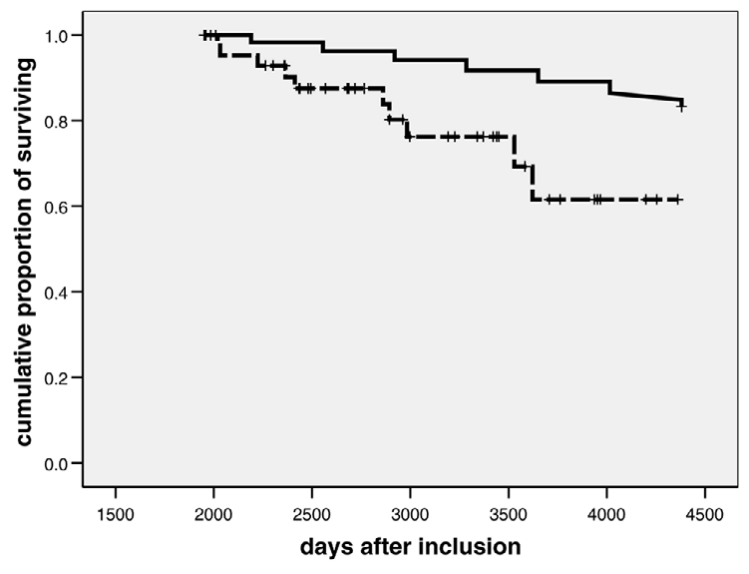
Twelve-year survival: patients who have already survived longer than five years versus general population. Presented are Kaplan-Meier plots showing 12-year survival rates (after inclusion) in patients having already survived for more than five years (dashed line) and in the German general population (continuous line; data from Statistisches Bundesamt Wiesbaden, Germany [30]). *P *< 0.001 versus reference population of 1,000 individuals

Unadjusted long-term survival rates after inclusion, after surgical ICU discharge, or after day 150 or year 5 after inclusion are presented in Table 2 and in Figures 2 and 3 (Kaplan-Meier analyses). There were no significant differences between male and female patients. After surgical ICU discharge, survival rates were persistently lower than those of the German general population. Similar results were obtained when long-term survival was analyzed in patients who survived for longer than 150 days after inclusion (Figure 2). In the latter subgroup five-year survival after inclusion was 55.7% and 12-year survival was 29.0%. Long-term survival rates were clearly less than predicted, and even in patients surviving more than five years life expectancy was significantly shorter that in the German general population (Figure 3).

**Table 2:** Long-term survival after more than 28 days of intensive care therapy or after ICU discharge and in age-matched German general population

Time of assessment	Survival			
	1 year	2 years	3 years	5 years

After day 28	33.0%	27.0%	23.9%	19.8%
After ICU discharge (male and female)	61.8%	50.6%	44.7%	37.0%
After ICU discharge (male)	64.7%	53.8%	46.0%	37.7%
After ICU discharge (female)	53.8%	41.6%	41.6%	36.3%
General population (male, age 65 years)^a^	98.3%	96.5%	94.6%	90.3%
General population (female, age 65 years)^a^	99.2%	98.4%	97.4%	95.2%

^a^Data from Statistisches Bundesamt Wiesbaden, Germany [30].

### Effect of admission date on outcome

Because admission data could not be fitted by arithmetic transformations, these data were divided into two classes based on the median (before and after 1 March 1999). Crude ICU survival rates were comparable between the two time intervals (51.5% during the period from 1993 to 1999, and 54.7% during the period from 1999 to 2005; not significant). Correspondingly, there were no differences in long-term survival after inclusion. Unadjusted one-year, three-year and five-year survival rates after inclusion added up to 35.4%, 25.9% and 22.2% during the interval between 1993 and 1999, and to 30.5%, 21.6% and 13.7% during the interval between 1999 and 2005 (not significant, according to log-rank testing). Also, after adjusting for potential confounders, acute and two-year prognosis was not affected significantly by admission date (before or after 1 March 1999; Table 3). However, we observed a significant difference with respect to cause of death during surgical ICU stay. Single organ failure as the cause of death was significantly more common in patients dying before March 1999 (23.0%) than in those dying thereafter (9.9%; *P *< 0.05).

**Table 3:** Covariate-adjusted effect of admission date (before versus after 1 March 1999) on acute and long-term prognosis

Prognosis	HR)/OR (95% CI)	*P *value
Survival time until day 150 after inclusion	HR 1.206 (0.871–1.670)	0.260
Survival time until year 3 after inclusion in patients surviving > 150 days	HR 1.278 (0.653–2.500)	0.474
ICU mortality	OR 1.169 (0.551–2.481)	0.684
Two-year mortality rate in ICU survivors	OR 1.479 (0.773–2.829)	0.237

CI, confidence interval; HR, hazard ratio; ICU, intensive care unit; OR, odds ratio.

### Determinants of acute prognosis

**Figure 4:**
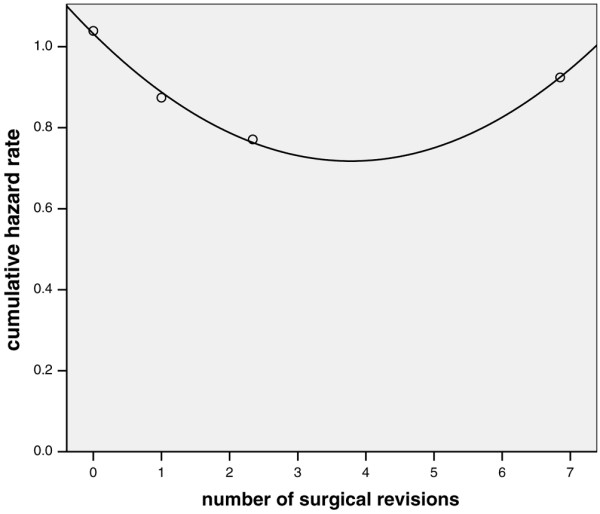
Univariate analysis of surgical efficacy versus cumulative hazard rate: first 150 days after inclusion. Shown is the univariate association between the number of surgical revisions (mean value per quartile) and the corresponding cumulative hazard rate for the first 150 days after inclusion. *P *< 0.001 after quadratic transformation of continuous data, and addition of a time-dependent covariate.

Multivariate analysis identified advanced age, duration of catecholamine therapy, surgery for thoracic diseases, peritonitis, maximum APACHE II score during the surgical ICU stay, and maximum number of failing organs as independently associated with ICU mortality (Table 4). The *P *value from Hosmer-Lemeshow statistical analysis was 0.935. With the exception of duration of catecholamine therapy, the same variables could be identified as independent risk factors for time to death until day 150 after inclusion (Table 5). Additional determinants were pneumonia and the number of surgical revisions. The latter variable had a complex independent association with survival time, with only low number of surgical revisions being associated with prolonged survival time (Figure 4).

**Table 4:** Independent risk factors for ICU mortality

	Odds ratio (95% confidence interval)	*P *value
Age (per year)^a^	26.730 (1.970–362.622)	0.014
Maximum APACHE II score (per point)	1.567 (1.200–2.047)	0.001
Duration of catecholamine therapy (per day)^b^	10.188 (2.789–37.215)	< 0.001
Maximum number of failing organs (per organ)	6.913 (1.356–35.244)	0.020
Surgery for thoracic diseases	3.651 (1.541–8.647)	0.003
Peritonitis	6.437 (3.068–13.505)	< 0.001

^a^After quadratic transformation. ^b^After logarithmic transformation. APACHE, Acute Physiology and Chronic Health Evaluation; ICU, intensive care unit.

**Table 5:** Survival time analysis until day 150 after inclusion (independent risk factors)

	Hazard ratio (95% confidence interval)	*P *value
Age (per year)^a^	3.213 (1.823–5.665)	< 0.001
Maximum APACHE II score (per point)^b^	15.311 (5.860–40.005)	< 0.001
Number of surgical revisions (per revision)^c^	1.381 (1.154–1.652)	< 0.001
Time-dependent covariate for number of surgical revisions	1.689 (1.189–2.400)	0.003
Maximum number of failing organs (per organ)^c^	1.664 (1.260–2.198)	< 0.001
Pneumonia	2.263 (1.225–4.180)	0.009
Time-dependent covariate for pneumonia	1.480 (1.121–1.954)	0.006
Surgery for thoracic diseases	1.975 (1.335–2.921)	0.001
Peritonitis	1.789 (1.278–2.504)	0.001

^a^After power transformation. ^b^After logarithmic transformation.^c^After quadratic transformation. APACHE, Acute Physiology and Chronic Health Evaluation.

### Determinants of two-year prognosis

**Figure 5:**
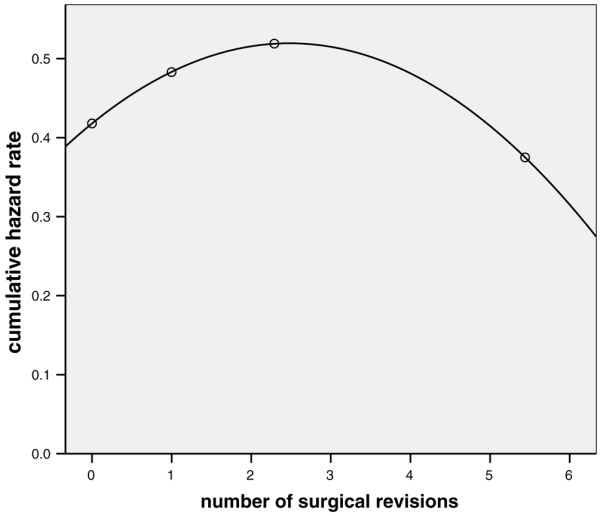
Univariate analysis of surgical efficacy versus cumulative hazard rate: first two years after inclusion. Univariate association between the number of surgical revisions (mean value per quartile) and the corresponding cumulative hazard rate for the first two years after inclusion in patients surviving more than 150 days. *P *= 0.033 after quadratic transformation of continuous data.

Variables that were independent determinants of two-year mortality in ICU survivors were advanced age, surgery for thoracic disease and palliative surgery for malignant disease (Table 6). *P *value from Hosmer-Lemeshow statistical analysis was 0.944. Advanced age and surgery for thoracic disease were also independent risk factors for a shorter survival time in patients surviving more than 150 days, as were surgery for malignant diseases, duration of mechanical ventilation (> 50 days), and the number of surgical revisions (Table 7). Again, a complex interaction of the latter variable with survival time was found, in which a lower number of surgical revisions was associated with a shorter survival time (Figure 5).

**Table 6:** Independent risk factors for two-year mortality in ICU survivors

	Odds ratio (95% confidence interval)	*P *value
Age (per year)^a^	25.524 (1.495–435.670)	0.025
Surgery for thoracic diseases	3.004 (1.223–7.379)	0.016
Palliative surgery	23.863 (3.098–183.788)	0.002

^a^After quadratic transformation. ICU, intensive care unit.

**Table 7:** Survival time analysis until the third year after inclusion (independent risk factors) in patients surviving more than 150 days

	*P *value	Hazard ratio (95% confidence interval)
Age (per year)	0.019	1.044 (1.007–1.083)
Time-dependent covariate for age	0.028	0.949 (0.905–0.994)
Duration of mechanical ventilation^a^	0.007	2.306 (1.250–4.254)
Palliative surgery	< 0.001	4.458 (2.032–9.778)
Number of surgical revisions (per revision)^b^	0.005	0.097 (0.019–0.495)
Surgery for malignant diseases	0.010	2.339 (1.227–4.460)

^a^For patients ventilated for more than 50 days. ^b^After quadratic transformation.

## Discussion

### Magnitude of short-term and long-term survival

Our analysis is the largest to describe the determinants and secular trends in acute and long-term mortality over a 12-year period in postoperative patients with an ICU length of stay of more than 28 days. We found that short-term prognosis in this particular patient group is limited (ICU survival rate 53.6%, 150-day survival rate after inclusion 42.3%). However, after successful surgery and intensive care therapy, long-term outcome in survivors is reasonably good, with five-year survival rates varying between 37% (in ICU survivors) and 56% (in patients surviving more than 150 days).

Acute survival rates in our mixed surgical cohort correspond well with those found by others in abdominal surgical, cardiac surgical, or mixed surgical/medical patients with a similar length of ICU stay [14,16,18,31]. Acute prognosis after chronic critical illness was only different among patients who were clearly younger [17,32,33] or older [15] than ours. Also one-year survival rate in our ICU survivors was almost similar to that found by other investigators in patients at a similar age [14,16] and was superior to that in older patients [15]. However, three-year and five-year survival rates in our cohort were about 10% lower than those seen after exclusively abdominal surgery [16] or in predominantly medical ICU patients [14] with a prolonged ICU length of stay and of similar age. The most likely explanation for this difference resides in the greater percentage of patients in our cohort who were suffering from malignant pulmonary diseases or had undergone palliative surgery. Both conditions may be expected to be associated with a less favourable long-term prognosis.

Compared with the general population, long-term survival rates after successful initial therapy were consistently lower in our patients, even beyond the fifth year. It is commonly believed that it may take 4 years or more for survival of ICU patients to parallel that in the general population [13]. However, this finding may only be valid for ICU populations with minor pre-existing illnesses. It is likely that independent effects of the primary disease process will be more important to long-term prognosis in surgical patients, who intrinsically suffer from major illnesses before the surgery. These diseases are the reason for the surgical intervention, and may only be superimposed temporarily by subsequent organ malfunction and consequent intensive care therapy.

### Prognostic factors

The extent to which the consequences of prolonged critical illness or treatments received in the ICU contribute to mortality, and whether these are potentially reversible, is still poorly understood. An expert panel convened by the European Intensive Care Society, the American Thoracic Society, and the Society of Critical Care Medicine [13] has identified late deaths after critical illness as a priority research area. The lack of long-term data compares unfavourably with what is known about the long-term course of other disease groups such as heart disease and cancer [13,34-36]. Therefore, one aim of our study was to identify prognostic factors that determine survival in patients with prolonged ICU stay.

Our analysis is the first to allow quantification of independent effects of the primary disease, severity of illness during ICU stay and treatments applied during intensive care. According to our findings, the greater case fatality rate in long-term survivors must predominantly be attributed to pre-existing diseases, especially malignancies, the presence of which was a strong determinant of long-term survival. Our analysis also shows that variables that relate to disease severity during the ICU stay or to ICU therapy have a rather important influence on acute survival (Tables 4 and 5), but they are of almost no importance to long-term survival (Tables 6 and 7). The validity of these findings is supported by the fact that almost identical results were obtained by two different statistical methods (logistic regression analysis and nonproportional hazard analysis of survival time).

Several important conclusions may be drawn from our multivariate analysis, and these are discussed below.

#### Age

Old age represents a strong independent risk factor for both poor acute and poor long-term prognosis after prolonged critical care. This nonlinear effect of age on patient prognosis is suggested by the fact that only a quadratic or power transformation of the age data yielded the necessary linear association between age and outcome in three of the four statistical models used (Tables 4, 5, 6). However, in long-term survivors (> 150 days after inclusion) the effect of age appeared to decrease over time, because the hazard ratio of the time-dependent covariate was under 1 (Table 7).

As was recently reviewed [13], age presumably influences long-term prognosis in critically ill patients to a large extent by being a marker for residual functional disability. However, it should be noted that, because of the nature of our study, patients with extreme physical disabilities were not included in our analysis. Because all of the patients included in the study had undergone elective or emergency surgery, their preoperative physical state must have been such that they were expected to survive at least the surgical procedure and the immediate postoperative phase.

#### Duration of mechanical ventilation

A particularly long duration of mechanical ventilation (> 50 days) was the only independent variable that was related to ICU therapy and was found to be associated with shorter long-term survival in patients surviving longer than 150 days. A worse long-term prognosis after prolonged invasive ventilation (> 49 days) was previously suggested by the univariate analysis conducted by Gracey and coworkers [14]. This interaction was elaborated by subsequent studies that adjusted for potential confounders when evaluating patients who needed invasive ventilation for longer than 21 or 35 days [37,38].

#### Effectiveness/efficacy of surgery

From the surgical perspective, there appears to be a fairly complex but significant association between surgical efficacy (as indicated by the number of surgical revisions) and outcome. Although the number of revisions was not associated with a significantly worse ICU or two-year survival by logistic regression analysis, it was a strong determinant of acute and long-term survival time (Figures 4 and 5). Thus, small numbers of surgical revisions lead to a longer survival time during the first months after inclusion but shortened survival time in patients surviving for longer than 150 days. On the other hand, a large number of surgical revisions (more than three or four) was not associated with a particularly poor or favourable acute or long-term prognosis. These findings may reflect a selection process in which a large number of re-operations is only possible in patients who are fit enough to withstand prolonged critical illness. Furthermore, these revisions will be only done in those patients judged likely to derive benefit from repeated interventions. However, we cannot completely exclude the possibility that those opposing effects on survival time were simply due to statistical heterogeneity and insufficient numbers of patients with multiple surgical revisions.

#### Initial severity of illness

APACHE II score in the first 24 hours after admission had no impact on acute or long-term prognosis in our patient cohort. The absence of an association between 24-hour APACHE II score and acute outcome after prolonged critical care was previously demonstrated [18,32]. The lack of influence of disease severity at admission on prognosis may once again suggest a selection process. Specifically, patients might not have not survived until week five either because they were too sick to respond to therapy or because they were among the ones who would have responded to therapy but did not receive appropriate treatment. Furthermore, patients with minor disease severity will already have left the surgical ICU by that time. Therefore, disease severity during the ICU stay appears to be much more important for acute prognosis than the initial extent of organ dysfunction.

#### Catecholamine therapy

Duration of catecholamine therapy was an independent prognostic determinant only when ICU survival was analyzed. This acute effect corresponds to observations by others who also evaluated determinants of acute outcome in patients undergoing a very long stay in the ICU [18]. These findings emphasize the importance of ongoing circulatory failure (as reflected by the use of vasoactive drugs) to acute prognosis in prolonged critical illness.

#### Malignancy

In contrast to long-term prognosis, acute prognosis was not worsened by extended tumor disease. The lack of importance of tumour extent to acute survival has previously been demonstrated by several investigators and has stimulated the concept of conducting intensive care regardless of tumour stage [39,40]. It appears that even a prolonged ICU length of stay would not conflict with the application of such a concept during care in palliative patients.

### Secular changes

A further aim of our study was to examine whether implementation of recent advances in critical care medicine has improved prognosis in chronically critically ill patients in our institution. We found that acute and long-term outcome had remained unchanged between 1993 and 2005 in our patients. The only significant secular change concerned the importance of single organ failure, which was less often a cause of death after 1999. Thus, it appears that treatment of individual organ failure (for instance, therapy for pulmonary failure) became more effective during the period of observation than did therapy for multiple organ failure. However, improved control of severe single organ failure might have allowed more patients to develop multiple organ dysfunction in later years. Multiple organ failure represents a highly complex condition in which therapeutic targets may often conflict with each other, thereby possibly preventing secular improvement in survival. The lack of improvement in acute prognosis is at odds with the findings of a variety of other studies [1-10], but it presumably emphasizes the extraordinary circumstances that may be encountered in patients with prolonged critical illness. It should be noted that our analysis only allows recognition of a relative improvement in short-term survival rate by about 15% (absolute improvement in 150-day survival from 40% to 55%). Therefore, we cannot exclude minor advances in prognosis. Two hypotheses may be proposed to account for the unchanged prognosis in surgical patients following prolonged critical illness.

First, recent evidence-based recommendations for intensive care therapy (such as strict glycaemic control or use of low tidal volumes during mechanical ventilation) have been derived from studies of interventions designed to treat an acute life-threatening insult [19]. Patients who survive this initial intensive care period and remain in the ICU for prolonged periods of time (such as our cohort) may experience a second threat, which is likely to be related to the risks associated with the prolonged ICU stay and includes ventilator-acquired pneumonia, catheter or urinary tract infection, persistent abdominal septic foci, or multiple organ dysfunction. These secondary, recurrent threats may be much less susceptible to strategies developed to manage the initial insult and may ultimately kill the patient [41].

Second, it is possible that the acute survival benefit of evidence-based therapeutic strategies does not persist beyond hospital discharge. For example, analysis of the effect of drotrecogin alfa (activated) on long-term survival after severe sepsis demonstrated that treated patients had a higher survival rate at hospital discharge. However, there was no statistical difference between treatment arms in duration of survival or differences in survival rates at 3 months, 1 year and 2.5 years after discharge [11].

### Limitations of the study

The present study has a number of limitations. Besides the primary diagnosis, a key role for ICU outcome determination must be attributed to specific structures or process qualities. More than 20 variables, such as length of shifts for house officers and nurse/patient ratio, have been identified as independent determinants of patient outcome in the ICU [42]. Although during the 12-year study period structures or processes not directly related to specific technical aspects of therapy remained largely unchanged on our ICU, we cannot completely exclude an effect of these potential confounders on the results of our study.

A further bias relevant to investigations of patient mortality may arise from the individual preferences of the treating physicians to continue or withdraw life support after a certain duration of ICU therapy [43]. Although the same senior intensivists were in charge during the entire period of study, a constant albeit subjective attitude toward discontinuation of life supportive measures cannot always be guaranteed.

In addition, the results of our study may not be generalizable because they represent the experience of a single centre and reflect a unique organization and process of care. Because there were no medical ICU patients or patients, for instance after cardiac surgery or neurosurgery, our findings may not be entirely applicable to patient cohorts others than ours.

On the other hand, our crude findings regarding acute and one-year survival rates corresponded well with findings in other patient cohorts with a comparable ICU length of stay or age, but with different primary diagnosis [14,16,18,31]. Therefore, we feel that at least some conclusions of our study may also valid for unselected populations of ICU patients. Such general conclusions may especially pertain to categories of determinants that influence acute and long-term prognosis.

## Conclusion

Despite a high acute fatality rate, long-term prognosis in chronically critically ill surgical patients is reasonably good. However, it is not comparable to that of the general German population, even beyond the fifth year after inclusion. Acute survival is determined by disease severity during ICU stay and by pre-existing illnesses, whereas long-term survival mostly depends on the underlying disease. Older patients appear to be at a particularly high risk for death and shorter survival. Acute and long-term prognosis have not changed during the past 12 years.

## Key messages

• No change in acute and long-term survival of chronically critically ill surgical patients has occurred at our institution over the past decade.

• Disease severity during ICU stay is a strong determinant of acute prognosis, but it is of almost no importance to long-term prognosis, which is mainly determined by pre-existing illnesses.

• Long-term survival in chronically critically ill surgical patients is reasonably good after successful surgery and intensive care therapy, but it is not comparable to that in the general population.

## Abbreviations

APACHE = Acute Physiology and Chronic Health Evaluation; ICU = intensive care unit.

## Competing interests

The authors declare that they have no competing interests.

## Authors' contributions

WH designed the study and drafted the manuscript. CPS and HW participated in generating data. HK participated in the design of the study and performed the statistical analysis. KWJ conceived the study, participated in its design and coordination, and helped to draft the manuscript. All authors read and approved the final manuscript.
